# Neutrophil, neutrophil extracellular traps and endothelial cell dysfunction in sepsis

**DOI:** 10.1002/ctm2.1170

**Published:** 2023-01-11

**Authors:** Hao Zhang, Yanghanzhao Wang, Mengdi Qu, Wenqian Li, Dan Wu, Juan P. Cata, Changhong Miao

**Affiliations:** ^1^ Department of Anesthesiology Zhongshan Hospital Fudan University Shanghai China; ^2^ Shanghai Key laboratory of Perioperative Stress and Protection Shanghai China; ^3^ Department of Anesthesiology Shanghai Medical College Fudan University, Shanghai, China; ^4^ Department of Anesthesiology and Perioperative Medicine The University of Texas‐MD Anderson Cancer Center Houston Texas USA; ^5^ Anesthesiology and Surgical Oncology Research Group Houston Texas USA

**Keywords:** endothelial cell dysfunction, neutrophil, neutrophil extracellular traps, sepsis

## Abstract

Sepsis is a persistent systemic inflammatory condition involving multiple organ failures resulting from a dysregulated immune response to infection, and one of the hallmarks of sepsis is endothelial dysfunction. During its progression, neutrophils are the first line of innate immune defence against infection. Aside from traditional mechanisms, such as phagocytosis or the release of inflammatory cytokines, reactive oxygen species and other antibacterial substances, activated neutrophils also release web‐like structures composed of tangled decondensed DNA, histone, myeloperoxidase and other granules called neutrophil extracellular traps (NETs), which can efficiently ensnare bacteria in the circulation. In contrast, excessive neutrophil activation and NET release may induce endothelial cells to shift toward a pro‐inflammatory and pro‐coagulant phenotype. Furthermore, neutrophils and NETs can degrade glycocalyx on the endothelial cell surface and increase endothelium permeability. Consequently, the endothelial barrier collapses, contributing to impaired microcirculatory blood flow, tissue hypoperfusion and life‐threatening organ failure in the late phase of sepsis.

## Graphical abstract

1. Neutrophils show increased lifespan and impaired migration, both of which promote organ damage.

2. Neutrophils and neutrophil extracellular traps (NETs) induce pro‐inflammatory and pro‐angiogenic responses in endothelial cells.

3. Neutrophils and NETs degrade glycocalyx and increase endothelial permeability.

4. Neutrophils and NETs induce a pro‐coagulant endothelial cell phenotype via degradation of the anti‐coagulation system and up‐regulation of tissue factors.

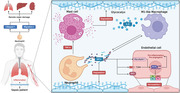

## 1 INTRODUCTION

Sepsis is defined as a lethal organ dysfunction resulting from a dysregulated immune response to infection. It still has a high rate of morbidity and mortality.^1^ During sepsis, neutrophils play a critical role in the host's inflammatory response against invading pathogens.^2^, ^3^ They exert effector functions primarily through three approaches: phagocytosis, degranulation and releasing neutrophil extracellular traps (NETs).^4^, ^5^, ^6^ NETs are extracellular, web‐like decondensed nuclear or mitochondrial DNA structures composed of histones, cytosolic and granule proteins with microbicidal activity.^7^, ^8^, ^9^ NETs neutralize and kill bacteria, fungi and viruses, and can inhibit their dissemination.^10^ However, if dysregulated, excessive NETs can further induce inflammation and organ injury contributing to the progression of sepsis.^11^, ^12^ Other immune cells, such as macrophages and eosinophils, can also release extracellular traps (ETs) that result in the killing of pathogens and participate in sepsis and other diseases, such as autoimmune processes.^13^, ^14^

During sepsis, neutrophils display increased lifespan and impaired migration. This confines them to blood vessels and results in overwhelming vascular inflammation via the release of cytokines, reactive oxygen species (ROS) and NETs.^15^, ^16^ Notably, neutrophils and NETs stimulate pro‐inflammatory and pro‐angiogenic responses in endothelial cells that cause further dysregulation of the immune system.^17^, ^18^ Activated endothelial cells display an increased glycolysis rate, further promoting inflammation and oxidative stress.^19^, ^20^ Direct destructive effects from NET components, accompanied by an inflammatory environment and oxidative stress, will degrade glycocalyx existing on the surface of the endothelial cells and increase endothelial permeability via junction cleavage, high expression of adhesion molecules and apoptosis.^21^ Additionally, neutrophils and NETs induce a pro‐coagulant endothelial cell phenotype via degradation of the anti‐coagulation system and up‐regulation of tissue factor (TF).^22^, ^23^ Consequently, the collapse of the endothelial barrier enhances microvascular leakage that triggers vascular hypotension, tissue oedema and lethal organ failure in sepsis.^24^

In this review, we will focus our discussion on the role of neutrophils, NET formation and sepsis progression, and then summarize their impacts on endothelial dysfunction. Lastly, we will provide evidence on potential targets for therapy.

## 2 NEUTROPHIL, NET FORMATION AND SEPSIS

Neutrophils show a critical role in innate immunity since they are the first line of defence against pathogens.^25^ During infection or inflammation, neutrophils escape from the bone marrow in large numbers.^26^ This process is accompanied by releasing proteolytic enzymes, ROS and reactive nitrogen species (RNS).^27^, ^28^ Under normal conditions, neutrophils display the shortest lifespan of all leukocytes and experience regulated cell death, which is crucial for preventing sustained inflammatory responses and tissue repair.^29^, ^30^ During sepsis, most immune cells tend to undergo apoptosis forming an immunosuppressive environment, while neutrophils display delayed apoptosis, resulting in prolonged inflammation.^15^ Activation of several signal pathways explains neutrophil resistance to apoptosis. In the peripheral circulation, pro‐inflammatory factors like complement component 5a (C5a) and lipopolysaccharide (LPS) activate extracellular regulated protein kinases (ERK) 1/2 and phosphoinositide‐3 kinases (PI‐3K), which results in the phosphorylation of Akt and subsequent Bad phosphorylation. Finally, this prevents the formation of the apoptosome.^31^, ^32^ C5a also enhances the expression of anti‐apoptotic protein Bcl‐xL and reduces Bim expression, which further suppresses caspase, a terminal splicing enzyme essential for cell apoptosis. When activated by LPS, the myeloid nuclear differentiation antigen prevents proteasomal degradation of myeloid cell leukaemia‐1 (MCL‐1), an anti‐apoptotic factor of the Bcl‐2 family.^33^ In rodents, single‐cell RNA sequencing confirmed that after LPS stimulation, multiple subclusters of neutrophils were differentiated and the programmed cell death ligand 1 (PD‐L1) was highly expressed in a particular cluster.^34^ Mechanistically, sepsis can enhance PD‐L1 expression on neutrophils, triggering lymphocyte apoptosis via direct contact, and finally promoting sepsis‐induced immunosuppression.^35^

During the early stages of sepsis, neutrophils expressing CXC receptor 2 (CXCR2) are recruited from the blood to the infection site, responding to CXC ligand 2 (CXCL2).^36^ After migrating to the site of the infection, neutrophils release NETs, ROS and RNS to kill the pathogens. However, during severe sepsis, neutrophil migration is impaired.

One of the mechanisms involved in this process is the activation of toll‐like receptors (TLRs) expressed on neutrophils. Alves‐Filho et al. found that direct activation of TLR2 on neutrophils contributed to CXCR2 down‐regulation and chemotaxis impairment.^37^ Consequently, neutrophils aggregate in blood vessels, facilitating the spread of pathogens. Studies have demonstrated that CC receptor 2 (CCR2), primarily absent in neutrophils under physiological conditions, is expressed in circulating neutrophils through TLRs activation. CCR2 drives inappropriate infiltration of neutrophils into remote organs that produce CC ligand 2 (CCL2), further eliciting tissue damage in organs such as the lung, liver and kidney.^38^

It is well known that older septic patients have poor prognoses.^39^ Inflammed aged tissues show a high frequency of neutrophil reverse transendothelial migration (rTEM) into the circulation, further contributing to remote organ damage.^17^ Barkaway et al. demonstrated that aged mice showed high levels of rTEM driven by mast cell‐derived CXCL1. In this paradigm, the intensified endothelial atypical chemokine receptor 1‐CXCL1 promoted desensitization of CXCR2 on neutrophils and loss of their directional motility, which further contributed to vascular leakage and organ damage.^17^ Moreover, an increased NET release was also found in the models of aged mice, which might exacerbate inflammation.^40^

In 2004, Brinkmann et al. proved the ability of neutrophils to release NETs. The authors speculated that NET formation was an early event in cell death.^41^ NET release occurs after stimulation with interleukin‐8 (IL‐8), LPS, or phorbol‐12‐myristate‐13‐acetate and involves activation of protein kinase C (PKC). Nicotinamide adenine dinucleotide phosphate (NADPH) helps neutrophil elastase (NE) translocate from cytosolic granules to the nucleus, where it causes chromatin breakdown by cleaving histone.^42^ NADPH oxidase activated by PKC and Raf‐MEK‐ERK signalling pathways promotes ROS production, which leads to an increased calcium influx, peptidyl arginine deaminase 4 (PAD4) activation and histone citrullination. Myeloperoxidase (MPO) facilitates the breakdown of chromatin and nuclear envelope, and granular mixing in the NET vacuole. After intracellular NET formation (NETosis), mature NETs are extruded due to the rupture of the neutrophil outer membrane.^42^ Remijsen et al. found that the formation of NETs involved both NADPH‐oxidase‐mediated superoxide production and autophagy, and both took place independently of each other.^43^ However, the involvement of autophagy has been controversial. Simon et al. demonstrated that NET formation was not dependent on autophagy in both mouse and human cell lines.^44^

**FIGURE 1:**
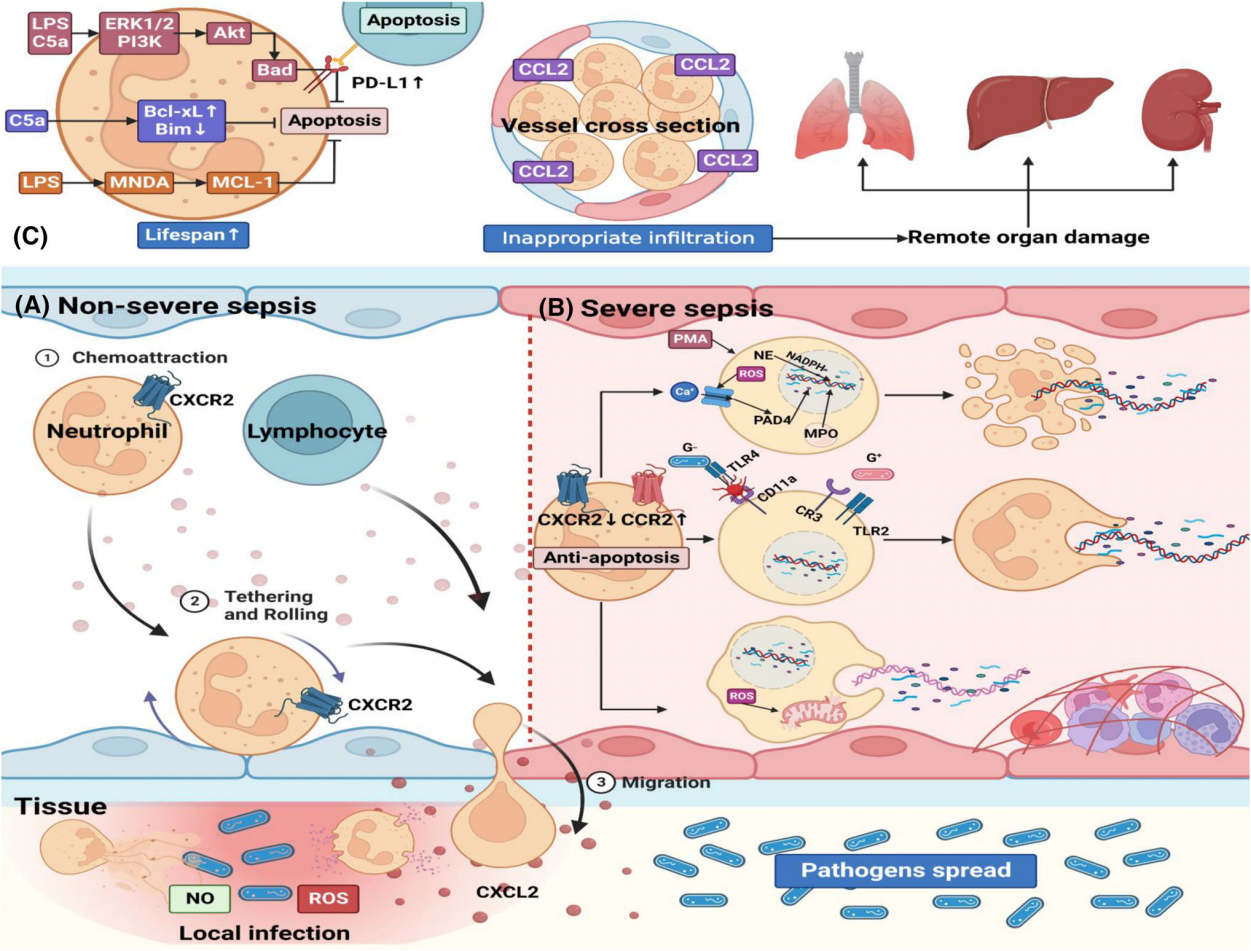
Neutrophil, NET formation and sepsis. (A) During non‐severe sepsis, neutrophils that express CXCR2 are recruited from blood to the infection site responding to CXCL2. Neutrophils migrate to the location of infection and kill pathogens through the release of antibacterial substances, such as ROS, NO and NETs. Other immune cells such as lymphocytes can also migrate to the infection site to prevent pathogens spread. (B) However, during severe sepsis, neutrophils show increased lifespan and impaired function due to the down‐regulation of CXCR2 and up‐regulation of CCR2. On the one hand, impaired migration results in pathogens' spread. On the other hand, many neutrophils are confined to vessels and release NETs, resulting in vascular inflammation, endothelial damage, and thrombosis. NET formation can be classified into three types. The first type is suicidal NETosis since NETs are released via cell lysis. The second type allows NET release and conventional live neutrophil functions, such as phagocytosis, to coexist. The third type is mtDNA NETosis. Viable neutrophils release mtDNA to form NETs, and this process does not depend on cell death but is dependent on ROS. (C) LPS and C5a in the peripheral circulation can induce neutrophils’ resistance to apoptosis via three signalling pathways. Moreover, neutrophils induce lymphocyte apoptosis via PD‐L1 up‐regulation. Additionally, CCR2, which is absent in neutrophils under normal conditions, is upregulated in neutrophils through TLR activation. CCR2 drives inappropriate infiltration of neutrophils into remote organs which produce CCL2 and further elicit tissue damage in remote organs such as lungs, liver, and kidneys. C5a, complement component 5a; CCL2, CC ligand 2; CCR2, CC receptor 2; CR3, complement receptor 3; CXCL2, CXC ligand 2; CXCR2, CXC receptor 2; ERK1/2, extracellular regulated protein kinases 1/2; G‐, gram‐negative bacteria; G+, gram‐positive bacteria; iNOS, inducible nitric oxide synthase; LPS, lipopolysaccharide; MCL‐1, myeloid cell leukemia‐1; MNDA, myeloid nuclear differentiation antigen; MPO, myeloperoxidase; mtDNA, mitochondrial DNA; NE, neutrophil elastase; NETs, neutrophil extracellular traps; PAD4, peptidyl arginine deaminase 4; PD‐L1, programmed cell death ligand 1; PI‐3K, phosphoinositide‐3 kinases; PI3Kγ, phosphoinositide‐3 kinase gamma; PMA, phorbol 12‐myristate 13‐acetate; ROS, reactive oxygen species; TLR, Toll‐like receptor

After direct interaction between neutrophil and platelet through CD11a, gram‐negative bacteria induce NET formation via TLR4 activation in platelets.^45^ Gram‐positive‐induced NETosis requires TLR2 and complement receptor 3.^46^ Aside from suicidal NETosis as noted above, neutrophils are capable of releasing NETs without membrane rupture. During NET production, vesicles of DNA, like beads on a string, bud from the nuclear envelope, pass through the cytoplasm and fuse with the outer membrane. Finally, NETs are transported out of the cell.^42^ This type of NETosis is ROS‐independent and requires a short period (30 min), while suicidal NETosis takes hours.^42^ It has been suggested that neutrophils could release mitochondrial DNA (mtDNA) in a cell death‐independent process.^47^ mtDNAs do not contain histones and are similar to plasmid DNA. After transporting nuclear DNA and mtDNA out of the cell, neutrophils are still equipped with effector functions, like chemotaxis, recruitment and phagocytosis (Figure 1).

As previously indicated, NETs play a pivotal role in host immunity. LL‐37, an antibacterial protein externalized on NETs, improved septic mice survival, possibly by preventing the release of pro‐inflammatory cytokines.^48^ However, the excessive release of NET promotes inflammation. In the extracellular space, host cell‐free DNA can serve as a damage‐associated molecular pattern (DAMP).^49^, ^50^ Also, mtDNA stimulates the secretion of tumour necrosis factor (TNF‐α) and IL‐1β from mouse splenocytes and macrophages, respectively.^51^ Extracellular histones are also considered DAMPs since they activate immune cells via TLR and nod‐like receptor signalling pathways. Xu et al. demonstrated that histones contributed to high levels of IL‐6, IL‐10 and TNF‐α.^52^, ^53^, ^54^

## 3 NEUTROPHIL, NETs AND IMMUNOTHROMBOSIS

Engelmann et al. reviewed the mechanism of NET‐mediated thrombosis, coining this process “immunothrombosis.” Although this process can ensnare pathogens and prevent dissemination, excessive thrombosis may further contribute to sepsis progression.^55^ Within NETs, DNA fibre and histone networks provide a scaffold to recruit erythrocytes, platelets, leukocytes and plasma proteins, thus forming a positive feedback loop that augments thrombosis in vivo and ex vivo.^56^ A recent study demonstrates that extracellular DNA in NETs can interact with von Willebrand Factor (vWF) and further promote thrombus formation and inflammation.^57^, ^58^ Moreover, the inflammation of damaged endothelial cells propagates the release of acute phase reactants, such as vWF and cell‐surface adhesion receptors into the circulation.^59^ Von Brühl et al. showed that NETs promoted the interaction between factor XII (FXII) and neutrophils, and initiated the intrinsic coagulation pathway.^60^ Data from intravital microscopy in septic mice indicated that the collaborative interaction between histone H4 in NETs induced intravascular coagulation.^61^ Additionally, NETs contributed to thrombosis by restraining anticoagulants, like antithrombin (AT), activated protein C (APC) and TF pathway inhibitor.^62^

A recent study demonstrated that neutrophils could also release NETs carrying active TF.^63^ High levels of TF‐enriched NETs in sepsis patients might result in immunothrombosis and worse disease outcomes.^64^ Thrombosis induced by NETs contributes to organ ischemic damage and disseminated intravascular coagulation (DIC).^65^ The rate of DIC is high in the late stage of sepsis and is linked to multiple organ failure and refractory septic shock.^66^

## 4 NEUTROPHILS AND NETs INDUCE A PRO‐INFLAMMATORY AND PRO‐ANGIOGENIC ENDOTHELIAL CELL PHENOTYPE

During sepsis, NETs trigger activation of the endothelium and may impair its structure and/or function.^18^ Engagement of pathogen recognition receptors, such as TLRs, drives endothelial cells to reprogram toward a pro‐inflammatory and pro‐angiogenic phenotype.^21^

Wojciak‐Stothard et al. found that markers of NETosis were increased in patients with pulmonary hypertension. The authors demonstrated that NETs could induce pro‐inflammatory and pro‐angiogenic responses in human pulmonary artery endothelial cells (HPAECs) via MPO/H_2_O_2_‐dependent activation of TLR4/NF‐κB signalling. They also showed that NETs markedly induced the expression levels of intercellular adhesion molecule‐1 (ICAM‐1), and several pro‐angiogenic factors, such as platelet‐derived growth factor (PDGF) and heparin‐binding EGF‐like growth factor in HPAECs.^18^

Activation of NF‐κB signalling enhances pro‐inflammatory and pro‐angiogenic responses in endothelial cells via increased expression of vascular cell adhesion molecule (VCAM‐1), platelet endothelial cell adhesion molecular‐1 (PECAM‐1) and elevated secretion of IL‐6, IL8 and vascular endothelial growth factor (VEGF).^67^ It has been reported that NETs induce neutrophil effector functions, such as exocytosis, ROS production and NET formation. These occur through several pathways related to the phosphorylation of Akt, ERK1/2 and p38.^10^ Interestingly, the production of IL‐8 is strongly linked to NET formation via mitogen‐activated protein kinase (MAPK) pathway activation.^68^

NETs induce an “M1‐like” macrophage phenotype characterized by the release of inflammatory cytokines like IL‐1, IL‐6, IL‐8 and TNF in vitro and in vivo.^69^ M1 phenotype macrophages also secrete several pro‐angiogenic factors, including VEGF and fibroblast growth factor (FGF).^70^ VEGF, as one of the most important growth factors, is capable of stimulating glycolysis via increased expression levels of glucose transporter 1 (GLUT1), fructose‐2,6‐bisphosphatase‐3 (PFKFB3) and lactate dehydrogenase‐A (LDH‐A).^71^, ^72^, ^73^, ^74^ In addition, activated VEGFR2 stimulates signalling via PI3K/Akt pathway. It thus suppresses the expression of growth‐inhibiting transcription factor *FOXO1*, which maintains endothelial cell quiescence through inhibiting glycolysis and mitochondrial respiration.^75^, ^76^ The growth‐enhancing transcription factor *MYC* resides in the nuclei of endothelial cells to drive the expression of genes that cooperatively facilitate proliferation and glycolysis.^75^

A previous study demonstrated that NE, as one of the components of NET, participated in the proteolytic cleavage of some growth factors crucial for angiogenesis, such as VEGF and PDGF.^77^ Ebos and Kerbel argued that the blockade of one growth factor could compensatorily upregulate the expression of others and stimulate angiogenesis again.^78^ This could explain why the efficacy of anti‐VEGF treatments varied among patients and resulted in only partial vessel regression.^79^ In animal models of inflammation, Mirabelli et al. reported that topical anti‐VEGF treatment reduced corneal neovascularization by only 14%.^80^ Mechanistically, when VEGF is blocked, the expression of other growth factors like FGF may be elevated, thus promoting glycolysis again through the FGF‐*MYC*‐HK2 axis.^81^ In the early phase of sepsis, various pro‐inflammatory cytokines and growth factors are overwhelmingly released; therefore, endothelial cells still retain a proliferative state characterized by high levels of glycolysis.

**FIGURE 2:**
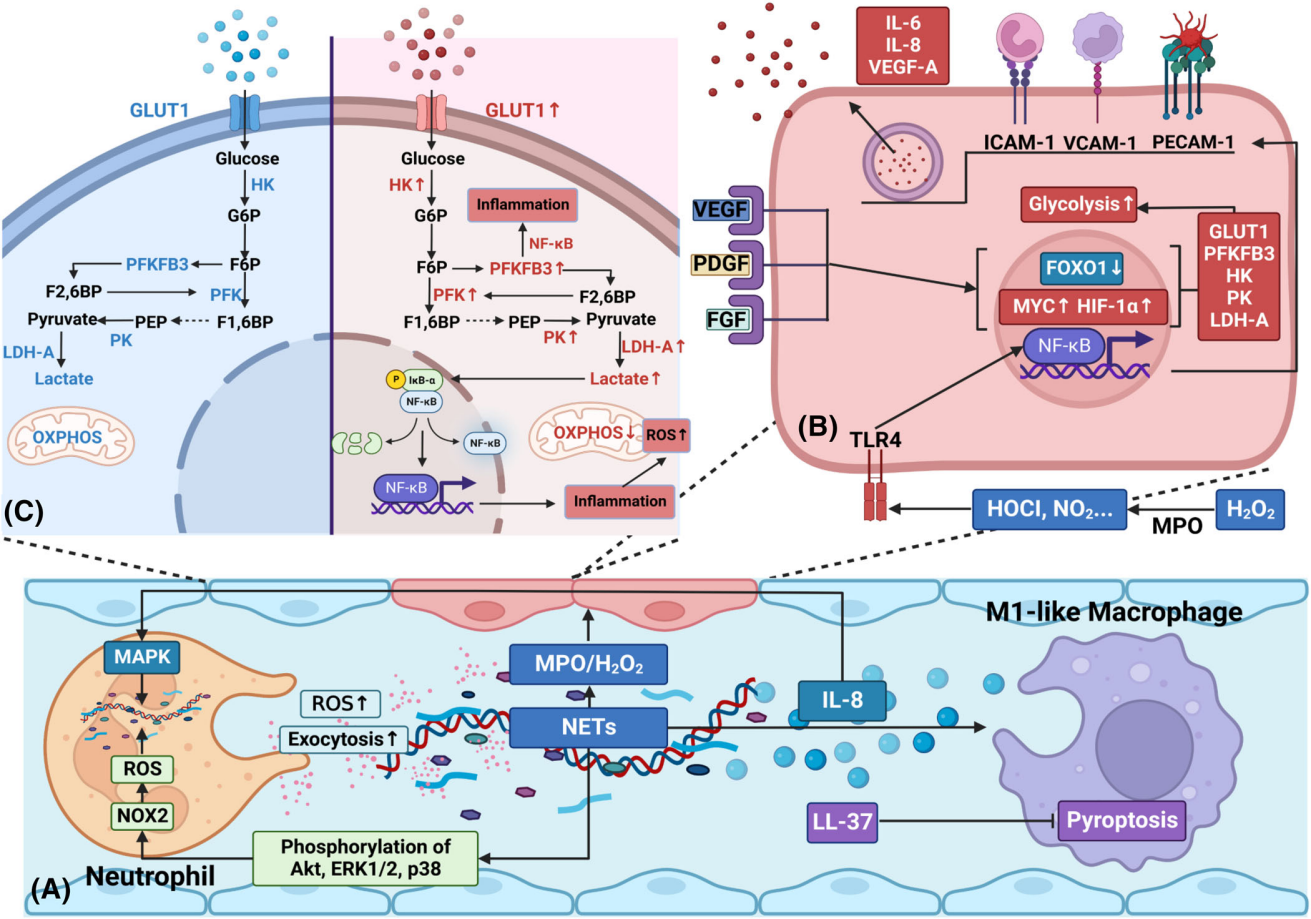
Neutrophils and NETs induce a pro‐inflammatory and pro‐angiogenic endothelial cell phenotype via activation of NF‐κB signalling. (A) NETs stimulate pro‐inflammatory and pro‐angiogenic responses in human endothelial cells via MPO/H_2_O_2_‐mediated NF‐κB activation. Moreover, NETs activate several neutrophil functions, such as exocytosis, ROS production, and NET formation, which may be related to the phosphorylation of Akt, ERK1/2 and p38. In addition to the impacts on neutrophils, NETs induce an “M1‐like” macrophage phenotype characterized by releasing inflammatory cytokines, such as IL‐8. Moreover, the concentration of IL‐8 is found to be strongly linked with NET formation via MAPK pathway activation. LL‐37, an antibacterial protein externalized on NETs, can suppress macrophage pyroptosis and then inhibit the release of pro‐inflammatory cytokines. (B) NF‐κB signalling enhances endothelial cell pro‐inflammatory and pro‐angiogenic responses via up‐regulation of ICAM‐1, VCAM‐1, PECAM‐1, and increased secretion of IL‐6, IL‐8, and VEGF‐A. Additionally, many pro‐angiogenic factors, such as VEGF, PDGE and FGF, are released during the process. These factors increase expression levels of glycolytic enzymes and transporters via up‐regulation of *MYC* and HIF‐1α and down‐regulation of *FOXO1*. (C) During inflammatory and angiogenic responses, proliferated endothelial cells show a higher rate of glycolysis than quiescent ones due to the up‐regulation of glycolytic enzymes and transporters, such as GLUT1, PFKFB3, HK, PK and LDH‐A. Notably, PFKFB3 and glycolytic product lactate promote NF‐κB‐driven vascular inflammation. F1,6BP, fructose‐1,6‐biphosphate; F2,6BP, fructose‐2,6‐biphosphate; F6P, fructose‐6‐phosphate; FGF, fibroblast growth factor; G6P, glucose‐6‐phosphate; GLUT1, glucose transporter 1; HIF‐1α, hypoxia‐inducible factor‐1α; HK, hexokinase; ICAM‐1, intercellular adhesion molecule‐1; LDH‐A, lactate dehydrogenase‐A; MAPK, mitogen‐activated protein kinase; NF‐κB, nuclear factor‐κB; NOX2, NADPH oxidase 2; OXPHOS, oxidative phosphorylation; PDGF, platelet‐derived growth factor; PECAM‐1, platelet endothelial cell adhesion molecule‐1; PEP, phosphoenolpyruvate; PFK‐1, phosphofructokinase‐1; PFKFB3, fructose‐2,6‐bisphosphatase‐3; PK, pyruvate kinase; VCAM‐1, vascular cell adhesion molecule‐1; VEGF, vascular endothelial growth factor

Notably, enhanced glycolysis promotes NF‐κB‐driven endothelial inflammation. Increased activity of PFKFB3 stimulates the expression of ICAM‐1 and VCAM‐1 in vitro and in models of acute lung injury. This appears to occur via NF‐κB activation, which further induces neutrophil and macrophage recruitment.^82^ Proliferating endothelial cells display high levels of glycolysis even in the presence of oxygen, similar to the Warburg effect observed in tumour cells.^83^ A similar metabolic shift in cancer vasculature can serve to explain changes in the metabolism of endothelial cells in sepsis. During tumour angiogenesis, lactate, the end‐product of glycolysis, activates NF‐κB by promoting the phosphorylation and degradation of the NF‐κB inhibitor IκB‐α.^19^ Subsequently, activated NF‐κB enhances endothelial cell pro‐inflammatory responses, as noted above.^84^, ^85^ All of these changes aggravate inflammation and oxidase stress during sepsis (Figure 2).

In short, neutrophils and NETs can induce pro‐inflammatory and pro‐angiogenic responses in endothelial cells via NF‐κB activation. Enhanced glycolysis in endothelial cells drives pro‐inflammatory programs and ROS accumulation. Consequently, this forms a vicious cycle that contributes to sustained vascular inflammation and overwhelming oxidative stress.

## 5 NEUTROPHILS AND NETs DAMAGE ENDOTHELIAL CELLS’ GLYCOCALYX AND INCREASE ENDOTHELIAL PERMEABILITY

As previously mentioned, neutrophils and NETs damage endothelial cells’ glycocalyx and increase endothelial permeability.^21^ This phenomenon may further contribute to dysregulated inflammation, impaired microcirculatory blood flow, tissue hypoperfusion and life‐threatening organ failure.^24^

The matrix meshwork covering the endothelial cells is glycocalyx, which consists of glycosaminoglycans (GAG), proteoglycans and glycoproteins. The GAG chains contain heparan sulfate, chondroitin sulfate and hyaluronic acid that binds to CD44. Selectins and integrins are glycoproteins that participate in neutrophil adhesion and coagulation.^21^ The endothelial glycocalyx also tethers the extracellular superoxide dismutase (SOD3), which prevents oxidative stress.^86^ The glycocalyx is also found in the intercellular spaces and on the side of endothelial cells close to the basal membrane. Damage to glycocalyx will cause an impaired vascular barrier and elevated microvascular permeability.^87^ As a result of glycocalyx disruption, endothelial cell adhesion molecules are exposed, which triggers further inflammation, rolling and adhesion of leukocytes and platelets.^88^

A clinical study demonstrated that pediatric trauma patients with high levels of histone‐complexed DNA (hcDNA) showed elevation in plasma levels of syndecan‐1 (a proteoglycan), suggesting the degradation of the glycocalyx.^89^ Enzymatic digestion by MPO, matrix metalloproteinase (MMP) and other NET‐containing proteinases can also induce glycocalyx degradation.^86^, ^90^ While the role of MMP‐9 activity is controversial, a clinical trial showed that the active form of MMP‐9 was only present in patients with severe sepsis.^91^ Additionally, many inflammatory cytokines, such as TNF‐α, IL‐6 and IL‐8, induced by neutrophils and NETs can directly damage glycocalyx.^92^ Mast cells are stimulated by TNF‐α, and they can release cytokines, proteases, histamine and heparinase, contributing to further glycocalyx degradation.^93^ A recent study demonstrated that disintegrin and metalloproteinase 15 were upregulated during inflammation and they cleaved CD44 at the membrane‐proximal region, which disrupted the integrity of the endothelial glycocalyx.^94^ In addition, oxidative stress‐induced histone deacetylase can upregulate MMP expression and inhibit tissue inhibitors of MMPs (TIMP1 and TIMP3), further activating MMP2 and MMP9, promoting syndecan‐1 and SOD3 shedding from the endothelial cell surface, and subsequently result in derangement of the endothelial glycocalyx.^86^

Para‐endothelial permeability increases during sepsis mainly due to junction cleavage. There are two common types of junctions existing between endothelial cells: tight junctions (TJs) and adherens junctions. TJs contain occludins and claudins, which are anchored to the actin cytoskeleton via zonula occludins (ZO).^21^ TNF‐α can disrupt claudin‐5 at cell‐cell junctions of endothelial cells by activating the NF‐κB pathway.^95^ ROS causes rearrangement of occludin from intercellular junctions, thus promoting its dissociation from ZO‐1 and augmenting endothelial soluble permeability.^96^

Adherens junctions mainly comprise vascular endothelial (VE)‐cadherins, which are linked to the actin cytoskeleton through catenins.^21^ VE‐cadherin is easily cleaved by MMP because of its vulnerability to enzymatic degradation, and this causes further damage to the junction integrity.^97^, ^98^ During NETs‐induced inflammatory response, cAMP/Rac1 signalling is suppressed. This, in turn, stimulates Rho activity and kinases such as Src and Pyk2,^99^ both of which lead to VE‐cadherin phosphorylation, dissociation from catenin and endocytosis.^100^ Endocytosis of VE‐cadherin increases gaps between endothelial cells, exacerbating the permeability of the vascular barrier.^101^

ICAM‐1 expression is significantly upregulated during inflammation and angiogenesis. Several studies have suggested that this adhesion molecule may assume a significant role in regulating barrier integrity.^102^, ^103^ On the one hand, emerging evidence demonstrates that ICAM‐1 promotes phosphorylation of VE‐cadherin, which in turn disrupts endothelial permeability in a neutrophil‐dependent fashion.^104^ On the other hand, Sumagin et al. suggested that ICAM‐1 overexpression increased endothelial permeability via PKC‐dependent signalling in the absence of leukocytes.^105^ Research also suggests that ICAM‐1 signalling can participate in transcellular permeability regulation, aside from its key role in regulating paracellular permeability. For example, caveolae are one of the main mechanisms of transporting albumin from the luminal side of the endothelium to the basement membrane. Importantly, Src phosphorylation of caveolin‐1 is facilitated by ICAM‐1 ligation.^21^

**FIGURE 3:**
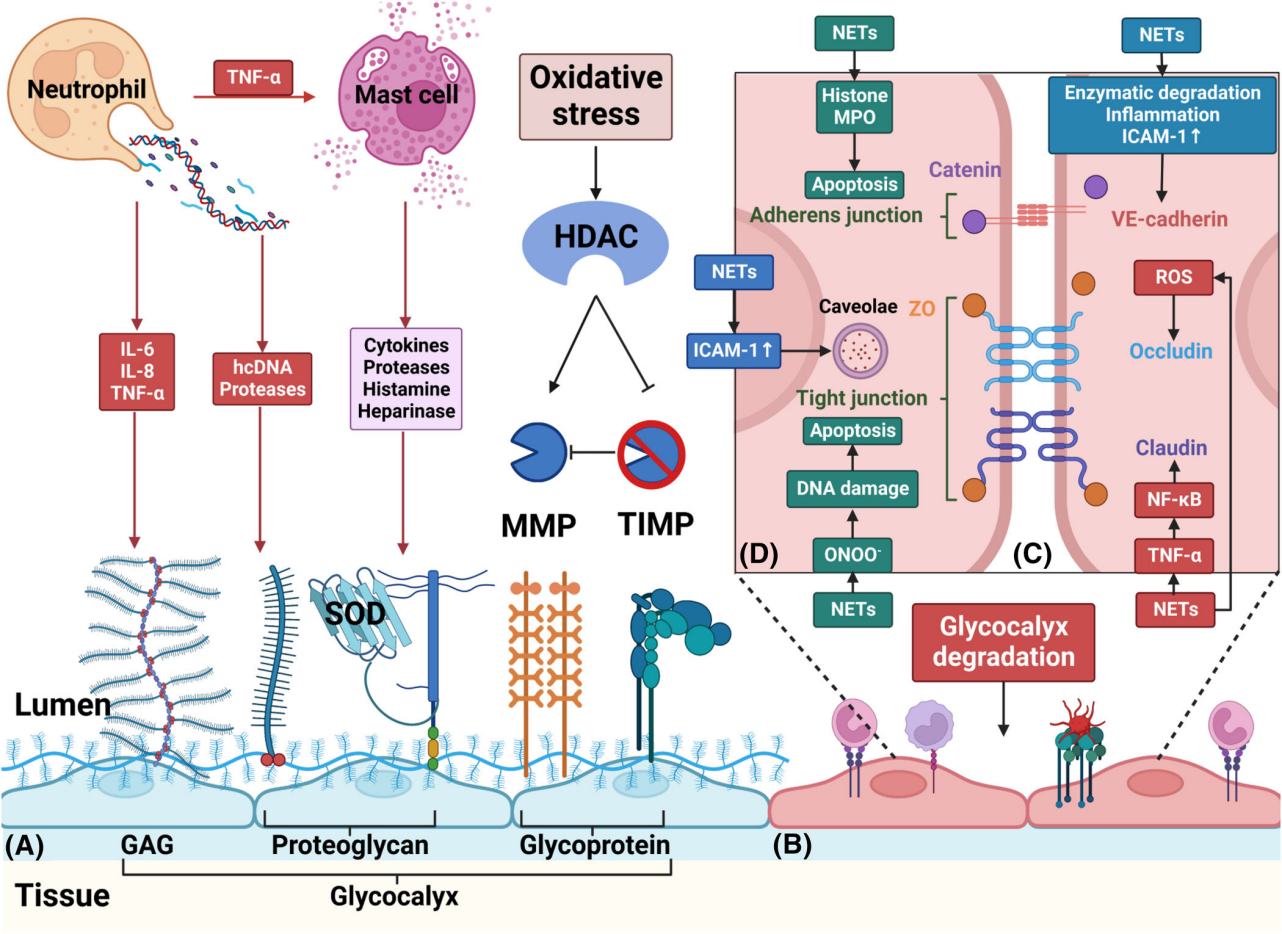
Neutrophils and NETs damage endothelial cells’ glycocalyx and increase endothelial permeability. (A) During sepsis, NETs can directly cause glycocalyx degradation through hcDNA and proteases. Additionally, a considerable number of inflammatory cytokines, such as IL‐6, IL‐8 and TNF‐α, induced by neutrophils and NETs can also damage glycocalyx. Moreover, mast cells are activated by TNF‐α and release cytokines, proteases, histamine and heparinase, further degrading glycocalyx. Furthermore, oxidative stress‐induced HDAC can upregulate MMP expression and inhibit tissue inhibitors of MMPs (TIMP1 and TIMP3), thereby activating MMPs to promote the shedding of the glycocalyx. (B) After degradation of the glycocalyx, endothelial cell adhesion molecules are exposed, triggering further inflammation, rolling, and adhesion of leukocytes and platelets. (C) Para‐endothelial permeability increases mainly due to junction cleavage. TNF‐α is shown to cause disruption of claudin through NF‐κB activation. ROS causes the redistribution of occludin, limiting its association with ZO‐1. VE‐cadherin is susceptible to enzymatic degradation. Additionally, inflammatory mediators promote VE‐cadherin phosphorylation and dissociation from p120 catenin and induce VE‐cadherin endocytosis via several signalling pathways. Upregulated ICAM‐1 can also promote VE‐cadherin phosphorylation. (D) ICAM‐1 can also increase trans‐endothelial permeability through caveolin‐1 phosphorylation, a major component of caveolae. However, endothelial cell apoptosis is the main cause of increased trans‐endothelial permeability. NETs contribute to apoptosis through MPO and histone. Moreover, the formation of ONOO^−^ during oxidative stress also induces cell death via DNA damage. GAG, glycosaminoglycan; hcDNA, histone‐complexed DNA; HDAC, histone deacetylase; MMP, matrix metalloproteinase; ONOO‐, peroxynitrite; SOD, superoxide dismutase; VE‐cadherin, vascular endothelial‐cadherin; ZO, zonula occluding

Endothelial cell apoptosis results in hyperpermeability.^24^ NETs promote endothelial apoptosis during sepsis. Saffarzadeh et al. found that NETs directly induced endothelial cell death and cytotoxicity partially mediated by histone and MPO.^106^ Furthermore, under pro‐inflammatory conditions, neutrophils and NETs trigger the production of O_2_^−^ and NO by 1000‐fold, increasing the formation of peroxynitrite (ONOO^−^) and causing a reduction in NO availability. Peroxynitrite induces permeabilization of the mitochondrial outer membrane, allowing the efflux of various pro‐apoptotic signalling molecules. Peroxynitrite is also capable of inducing DNA damage and then activates PARP‐1, a DNA repair enzyme. Upon severe DNA injury, excessive activation of PARP‐1 depletes the cellular stores of NAD^+^, a pivotal cofactor of the glycolytic pathway. Consequently, the loss of NAD^+^ results in the reduction of adenosine triphosphate (ATP), causing further endothelial dysfunction^107^ (Figure 3).

Neutrophils may also play a partial protective role in sepsis by reducing vascular leakage in some forms of pathogen‐specific infections.^108^, ^109^ For instance, during meningococcemia caused by Neisseria meningitides (Nm), neutrophil recruitment allows efficient phagocytosis, partially reducing vascular leakage induced by bacteria.^109^ Interestingly, Nm is a potent NET inducer. Lappann et al. found that NETs inhibited bacterial growth but could not exert killing activity.^110^

## 6 NEUTROPHILS AND NETS INDUCE A PRO‐COAGULANT ENDOTHELIAL CELL PHENOTYPE VIA DEGRADATION OF THE ANTI‐COAGULATION SYSTEM AND UP‐REGULATION OF TISSUE FACTOR

The glycocalyx covering the luminal side of the endothelium is extremely important in maintaining anti‐thrombogenicity in the vascular lumen. Once the glycocalyx is damaged by NETs, ICAM‐1, E‐selectin and other adhesion molecules are exposed to the denuded endothelium, which accelerates the recruitment of neutrophils and platelets.^111^ Additionally, GAGs prevent coagulation under physiological conditions.^112^ In the vasculature, heparan sulfate is the major component of GAGs, representing 50%‐90% of the total content.^113^ Heparan sulfate primarily binds to AT and dermatan sulfate to heparin cofactor II (HCII). Moreover, heparan sulfate can also interact with HCII. Antithrombin exerts its anticoagulant effects by inactivating both thrombin (factor IIa) and factor Xa, while HCII only inhibits thrombin.^114^, ^115^

TF also plays a vital role in coagulation. This protein is normally expressed on endothelial cells and is not in direct contact with the blood. When neutrophils and NETs damage the endothelium, TF is exposed and initiates plasma‐mediated hemostasis (via the so‐called extrinsic pathway). In this process, TF acts as a cellular receptor for plasma factor VIIa. Then, the complex of TF/VIIa activates factor IX and factor X. In an important reaction that requires factor V, factor Xa proteolytically cleaves prothrombin to thrombin. Finally, FXIIIa (a transglutaminase activated by thrombin) induces the formation of an insoluble fibrin clot.^116^, ^117^

NETs can also upregulate TF expression in endothelial cells. Folco et al. found that treating human endothelial cells with NETs increased the expression of TF mRNA and enhanced endothelial cell TF activity. Mechanistically, NETs induced TF production through IL‐1α and cathepsin G. This serine protease is abundant in NET and cleaves pro‐IL‐1α precursor, which then activates IL‐1α to promote TF production.^23^ Haubitz et al. showed that proteinase 3 (PR3) and elastase stimulated TF expression in human endothelial cells. While the elastase‐induced pro‐coagulant effect was blocked by α1‐antitrypsin (α1‐AT), suggesting that the effect depended on enzymatic activity. Interestingly, the pro‐coagulant effect of PR3 on endothelial cells was non‐enzymatic.^118^

Histones, another NET component, trigger an endothelial pro‐coagulant state via up‐regulation of TF and down‐regulation of the anticoagulant thrombomodulin (TM) in a process regulated by TLR2 and TLR4.^119^ Moreover, the cytotoxic effects of histone may trigger the exposure of phosphatidylserine (PS) on the endothelium. PS, normally existing on the inner layer of the plasma membrane, can stimulate the pro‐coagulant activity of TF by transferring it to the outer layer.^119^, ^120^ Hypochlorous acid, produced by NET component MPO,^121^ increases the levels of TF mRNA expression in human saphenous vein endothelial cells (HSVECs) and stimulates endothelial TF activity.^122^

A variety of anticoagulant mechanisms, especially the TM, protein C and endothelial protein C receptor (TM‐PC‐EPCR) anti‐coagulation pathway, are suppressed during sepsis or inflammation. The TM‐PC‐EPCR system is a crucial endogenous anti‐coagulation mechanism.^123^ During coagulation, thrombin and PC bind to their receptors, TM and EPCR, respectively, which form a thrombin‐TM‐PC‐EPCR complex on the endothelium. This further contributes to the formation of APC, a proteinase generated by thrombin from the zymogen precursor protein C.^124^ This proteinase has many anti‐coagulation properties, such as inactivating the procoagulant factors, FVa and FVIIIa and promoting the release of plasminogen activator.^125^

**FIGURE 4:**
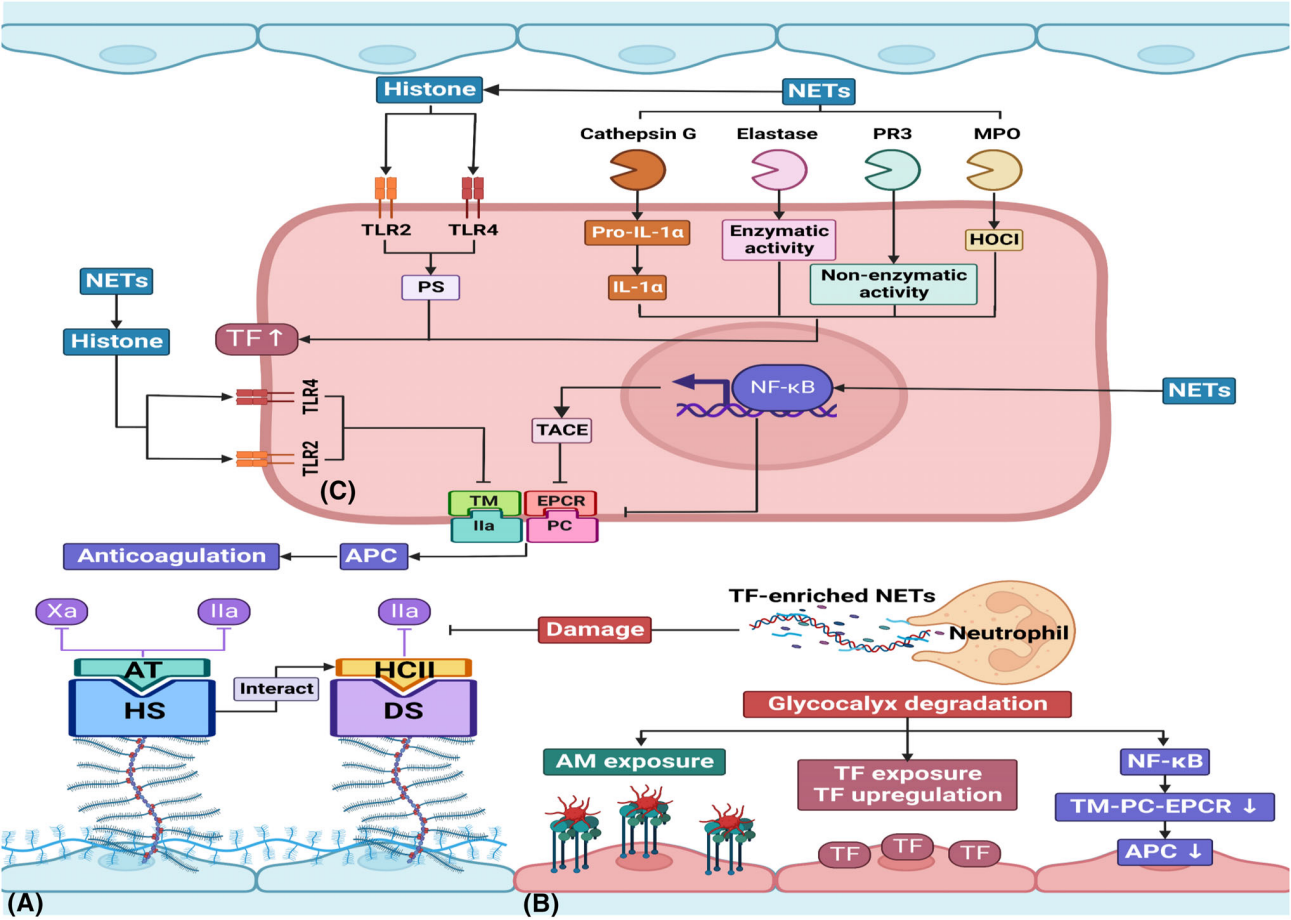
Neutrophils and NETs induce a pro‐coagulant endothelial cell phenotype via degradation of the anti‐coagulation system and up‐regulation of tissue factor. (A) GAGs play a key role in the anti‐coagulation system: HS primarily binds to AT to inhibit factor IIa and Xa, and DS mainly binds to HCII to inhibit factor IIa. Moreover, HS also interacts with HCII. So glycocalyx degradation induced by NETs contributes to the degradation of the anti‐coagulation system. (B) Furthermore, adhesion molecules and TF are exposed on the endothelium causing thrombus and fibrin formation. Aside from releasing TF‐enriched NETs, neutrophils increase expression levels of TF on endothelium through NET components. (C) Cathepsin G, elastase, PR3 and MPO can stimulate TF expression through different signalling pathways and further initiate the extrinsic coagulation pathway. Histone, another NET component, induces endothelial pro‐coagulant phenotype through up‐regulation of TF and down‐regulation of TM, and these effects are partly mediated by TLR2 and TLR4. Moreover, histone is capable of stimulating PS exposure, which enhances the pro‐coagulant activity of TF. Additionally, NETs can destroy the TM‐PC‐EPCR system, the most important natural anti‐coagulation mechanism, via NF‐κB activation. And NF‐κB signalling also promotes TACE activity, which is reportedly responsible for EPCR shedding. AM, adhesion molecule; APC, activated protein C; AT, antithrombin; DS, dermatan sulfate; HCII, heparin cofactor II; HOCI, hypochlorous acid; HS, heparan sulfate; PR3, proteinase 3; PS, phosphatidylserine; TACE, TNF‐α converting enzyme; TF, tissue factor; TM‐PC‐EPCR, thrombomodulin, protein C, and endothelial protein C receptor

As noted above, NETs can activate NF‐κB signalling in endothelial cells. This transcription factor has important functions in coagulation.^67^ Song et al. developed a transgenic mouse overexpressing mutant I‐κBα, an inhibitor of NF‐κB, on endothelial cells. Transgenic mice had a decreased level of thrombin‐AT, indicating that NF‐κB signalling was likely to participate in septic‐induced coagulation.^126^ Activation of endothelial‐specific NF‐κB signalling results in a decrease in cellular levels of TM and EPCR proteins in endothelial cells. Moreover, blocking endothelial NF‐κB signalling inhibits TNF‐α converting enzyme activity which is responsible for EPCR shedding, reducing plasma plasminogen activator inhibitor type‐1 level and restoring plasma level of APC^22^, ^127^ (Figure 4). Aside from its anticoagulant effects, APC also displays cytoprotective properties, such as blocking inflammatory cytokine release,^128^ maintaining endothelial integrity and protecting cells from apoptosis.^129^, ^130^ So decreased plasma levels of APC are strongly linked with prognosis. Although controversial, APC has been used in severe sepsis.^131^

## 7 POTENTIAL THERAPEUTIC TARGETS IN SEPSIS

### 7.1 Inhibition of NET formation in sepsis

**FIGURE 5:**
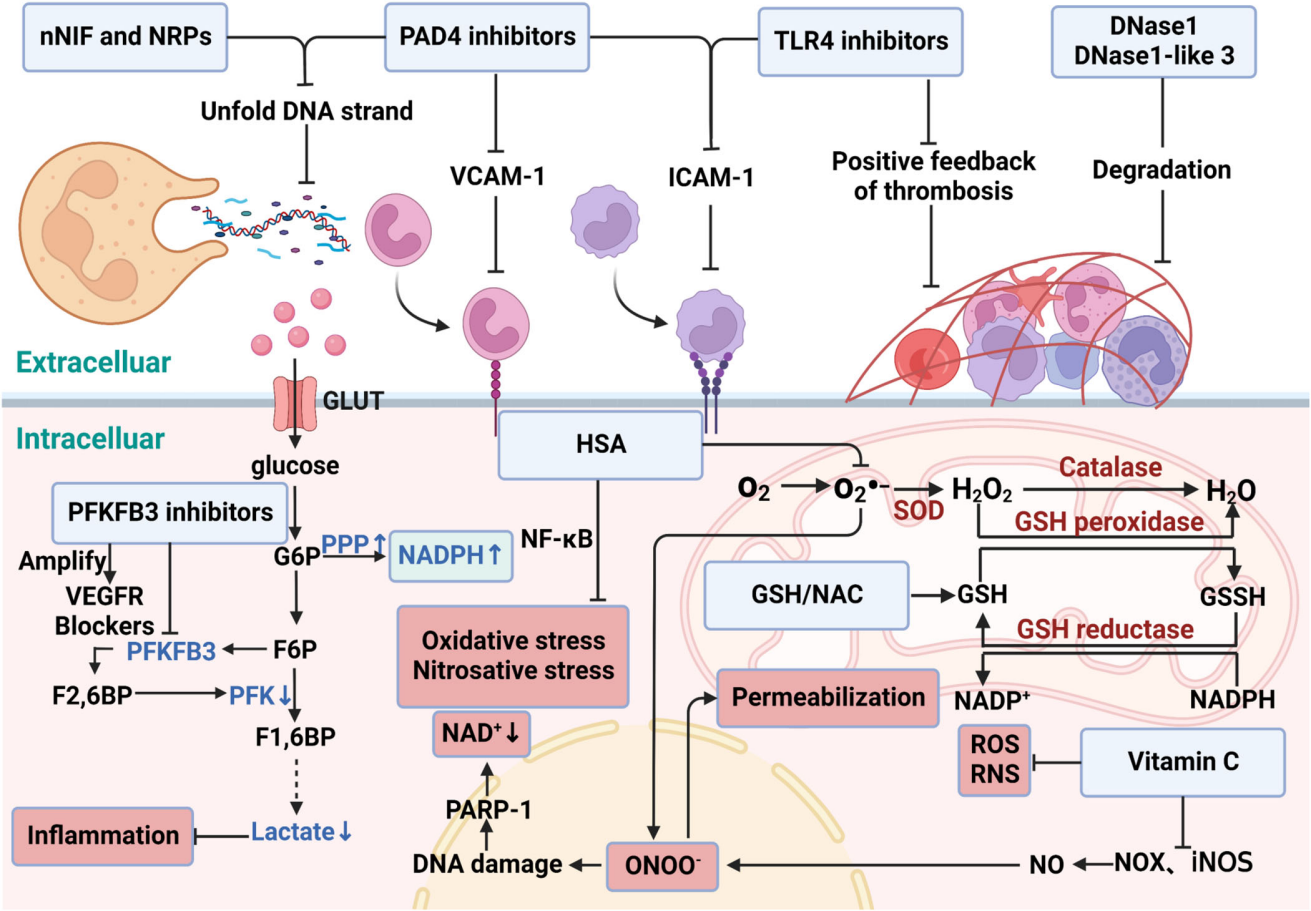
Potential therapeutic targets in sepsis. In blood vessels, nNIF, NRPs, and PAD4 inhibitors prevent NET formation by inhibiting chromatin decondensation. And PAD4 inhibitors may also reduce the expression of ICAM‐1 and VCAM‐1. TLR‐4^−/−^ mice did not show an enhanced thrombotic response and exhibited markedly decreased circulating ICAM‐1 compared with wide‐type controls. DNase1 and DNase1‐like 3 blocks the positive feedback loop of thrombosis by degrading the scaffold of NET structures. PFKFB3 blockers may be used to control inflammation induced by excessive glycolysis during sepsis. Using PFKFB3 inhibitors can also promote NADPH production, which is likely to induce NET formation. Moreover, PFKFB3 blockade amplifies the anti‐angiogenic effect of VEGFR blockers. In a perfused endothelial cell model, GSH or NAC (the precursor of GSH) supplementation significantly decreased ROS production. HSA can suppress circulating and tissue O_2_^−^ production, and restrain activation of NF‐κB, thereby inhibiting oxidative stress and nitrosative stress. In addition to scavenging ROS and RNS directly, vitamin C reduces them by preventing NOX activation, decreasing iNOS expression, and enhancing NO bioavailability. Then decreased formation of ONOO^−^ can efficiently prevent endothelial cell apoptosis. GSH, glutathione; HSA, human serum albumin; NAC, N‐acetylcysteine; nNIF, neonatal NET‐inhibitory factor; NRPs, nNIF‐related peptides; RNS, reactive nitrogen species

PAD4 is an important enzyme involved in chromatin decondensation. By catalyzing histone citrullination, PAD4 weakens the combination of DNA and histones (FIGURE 5 and TABLE 1).^7^ Overexpression of PAD4 contributes to severe vascular damage through the release of NETs and inducing expression of ICAM‐1 and VACM‐1 on endothelial cells.^23^ Therefore, using PAD4 inhibitors can prevent the NET formation and avoid endothelial cell dysfunction. Martinod et al. found that PAD4^−/−^ mice were partially protected from LPS‐induced shock.^132^ The PAD4 inhibitor Cl‐amidine effectively prevented NET formation and improved overall survival in a murine sepsis model.^133^ Inhibition of NET formation by the PAD4 inhibitor GSK484 led to an obvious reduction in thrombus formation in mouse lungs.^134^ The neonatal NET‐inhibitory factor (nNIF), can inhibit PAD4 activity, nuclear histone citrullination and nuclear decondensation. Yost et al. demonstrated that nNIF and nNIF‐related peptides could block NET formation in mouse models of infection and systemic inflammation.^135^ However, it is worth mentioning that PAD4 is not always required for NET release, since mtDNA is not equipped with histones, suggesting that its effects of inhibiting NET formation are limited.

**TABLE 1:** Potential targets for endothelial cell dysfunction therapy

						**Clinical trials**				
**Potential target**	**Mechanism**	**Compound**	**Effects**	**In vitro**	**In vivo**	I	II	III	IV	**Ref**
Inhibit NET formation	PAD4 inhibitor	Cl‐amidine	Effectively prevent the NET formation and improve overall survival in a murine sepsis model							^[^ ^127^ ^]^
		GSK484	Cause a dramatic reduction in thrombus deposition in mouse lungs							^[^ ^128^ ^]^
	TLR4 inhibitor	C34	Reduce systemic inflammation in the mouse models of endotoxemia							^[^ ^132^ ^]^
		TAK‐242	Suppress inflammation by combining with cysteine 747 existing in the TIR domain of TLR4, but fail to reduce cytokine levels in patients with sepsis							^[^ ^134^, ^135^ ^]^
		Eritoran	Inhibit LPS‐induced NF‐κB activation and inflammatory cytokine production in vitro and animal models, but do not reduce 28‐mortality among patients with severe sepsis							^[^ ^136^, ^137^, ^138^ ^]^
	Degrade DNA	DNase1	Preferentially cleave protein‐free DNA and degrade the scaffold of NET structures							^[^ ^143^ ^]^
		DNase1‐like 3	Preferentially cleave DNA‐protein complexes and degrade the scaffold of NET structures							^[^ ^143^ ^]^
	NET‐inhibitory factor	nNIF and NRPs	Block NET formation in vitro and mouse models of infection and systemic inflammation, and show improved prognosis in these models							^[^ ^144^ ^]^
Inhibit excessive glycolysis	PFKFB3 blockade	3PO	Protect mice from acute lung injury via suppression of NF‐κB‐mediated vascular inflammation							^[^ ^77^ ^]^
		PFK158	Suppress ATP production and proliferation in the mouse models of small cell lung cancer							^[^ ^149^ ^]^
		Phenoxyindole 44	A novel inhibitor of PFKFB3 with higher selectivity							^[^ ^150^ ^]^
Maintenance of the balance between ROS production and clearance	Antioxidant	GSH/NAC	Remarkably decrease ROS production when the cells were exposed to plasma from septic shock patients, impair NET formation in vitro and in vivo, contribute to shorter stays in ICU and improved severity scores							^[^ ^153^, ^154^, ^155^, ^156^ ^]^
		HSA	Reduce ROS and RNS production in vitro and in vivo, reverse sepsis‐induced hypotension in clinical trials							^[^ ^157^, ^158^ ^]^
		Vitamin C	Scavenge ROS and RNS, prevent NOX activation, and decrease expression of iNOS, but do not obviously improve organ dysfunction scores or change markers of vascular damage and inflammation among patients with sepsis							^[^ ^159^ ^–162]^

The green box denotes “Yes” and the red box denotes “No”.

TLR4 expressed on platelets is also responsible for NET induction in mice and humans.^45^ Obi et al. reported that TLR4^−/−^ mice did not show an enhanced thrombosis and exhibited conspicuously decreased circulating ICAM‐1 compared to wide‐type controls.^136^ Therefore, it suggested that inhibition of TLR4 could improve clinical outcomes in patients with sepsis.^137^ C34 is a novel inhibitor of TLR4 in vitro and in vivo. It reduces systemic inflammation in a mouse model of endotoxemia.^138^ TAK‐242 is another inhibitor of the TLR4 signalling pathway.^139^ This compound inhibits TLR4 signalling‐dependent inflammation by combining with cysteine 747 existing in the TIR domain of TLR4.^140^ Despite its promising laboratory results, a randomized controlled trial showed that TAK‐242 failed to reduce cytokine levels in patients with sepsis.^141^ Eritoran (E5564), a synthetic lipid A analogue of Rhodobacter aphaeroides, markedly inhibits LPS‐induced NF‐κB activation and inflammatory cytokine production in vitro and in vivo.^142^, ^143^ However, in a clinical study of severe sepsis, Eritoran did not reduce 28‐day mortality compared with a placebo.^144^ It should be mentioned that TLR4 inhibitors show indirect functions on NET formation and TLR inhibition might have pleiotropic effects.^145^ Claushuis et al. demonstrated that inhibition of TLR signalling in platelets did not alter the host immune response in murine sepsis models, arguing against the vital role of TLRs in sepsis progression.^146^

DNases block the positive feedback loop of thrombosis by degrading the scaffold of NET structures. DNase1 is expressed in nonhematopoietic tissues and preferentially cleaves protein‐free DNA.^147^ Moreover, immune cells secrete DNase1‐like3, which targets DNA‐protein complexes, such as nucleosomes.^148^ DNase1 and DNase1‐like3 also protect the endothelium during sepsis in vitro and in vivo.^149^

### 7.2 Inhibition of glycolysis in sepsis

Like tumour endothelial cells, activated endothelial cells are highly glycolytic during sepsis.^71^ Cantelmo et al. reported that tumour endothelial cells were more glycolytic than normal endothelial cells, as indicated by a high expression of PFKFB3. Therefore, the therapeutic concept of using PFKFB3 inhibitor has been extended to patients with sepsis. Furthermore, targeting endothelial cell glycolysis prevents pathological angiogenesis during inflammation.^150^ Wang et al. indicated that 3‐(3pyridinyl)‐1‐(4‐pyridinyl)‐2‐propen‐1‐one (3PO) treatment protected mice from acute lung injury via suppression of vascular inflammation.^82^ A new class of 3PO derivatives has also been synthesized to improve their anti‐angiogenesis properties. For example, PFK158, a small molecule inhibitor of PFKFB3, can suppress ATP production and proliferation in mouse models of small‐cell lung cancer.^151^ Additionally, phenoxyindole 44 shows a higher selectivity for PFKFB3 than the PFKFB1 and PFKFB2 isoforms.^152^

### 7.3 Maintenance of the balance between ROS production and clearance

During sepsis, an imbalance between ROS production and clearance is one of the main mechanisms of endothelial cell dysfunction. Glutathione (GSH) or *N*‐acetylcysteine (NAC, the precursor of GSH) remarkably decreased endothelial cell death and ROS production when endothelial cells were exposed to plasma from septic shock patients.^153^ Several clinical trials showed that patients treated with NAC had shorter stays in the ICU and improved sepsis severity scores.^154^, ^155^ Aside from clearing ROS, NAC was also shown to impair NET formation during bacterial infection, which could better explain the observed shorter stays and improved severity scores in ICU.^156^

Albumin has antioxidant effects associated with its thiol group. Meziani et al. used human serum albumin (HSA) in a rodent model of septic shock. The authors proved that animals treated with HSA showed a decrease in ROS and RNS production. HSA reduces circulating and tissue O_2_^−^ production, further preventing endothelial cell dysfunction. Indeed, a decrease in O_2_^−^ results in a reduction in its interaction with NO and thus suppresses ONOO^−^ production. Moreover, HSA inhibits the activation of NF‐κB signalling, thus reducing nitrosative stress and oxidative stress in the aorta.^157^

Finally, vitamin C has been used in patients with sepsis because of its antioxidant effect. After scavenging ROS and RNS, vitamin C is oxidized to ascorbate‐free radicals.^158^ Ascorbate further inhibits ROS and RNS production by preventing NOX activation, suppressing the expression of inducible nitric oxide synthase and enhancing NO bioavailability.^159^, ^160^ Unfortunately, a clinical trial showed that compared with a placebo, the infusion of vitamin C did not improve organ dysfunction scores or change markers of vascular damage and inflammation.^161^

## 8 CONCLUSION AND PERSPECTIVE

In sepsis, neutrophils and NETs constitute a robust antimicrobial defence. At the same time, excessive neutrophil activation and NET release convert endothelial cells from an anti‐inflammatory, anti‐coagulant phenotype to a pro‐inflammatory, pro‐coagulant phenotype. Additionally, NETs degrade glycocalyx and increase endothelial permeability. Endothelial cell barrier destabilization further promotes sepsis progression.

Thus far, several potential targets for endothelial cell dysfunction therapy have been proposed. Although it is increasingly recognized that excessive NET release may be a vital therapeutic target, studies have been inconclusive. Most inhibitors of NET formation are still in preclinical status, and further clinical trials are needed to be conducted. Anti‐glycolysis treatments are primarily used to inhibit tumour growth, and the therapeutic dosage and the time of administration to sepsis patients need further research. Notably, key enzymes in glycolysis, such as hexokinase, phosphofructokinase‐1 and pyruvate kinase, are also likely to be novel and promising therapeutic targets.

## FUNDING INFORMATION

This research was supported by the National Natural Science Foundation of China (NO. 82102253), Natural Science Foundation of Shanghai (NO. 21ZR1413400), Shanghai Sailing Program (NO. 21YF1406800) and the Shanghai Municipal 2021 “Science and Technology Innovation Action Plan” (NO. 21JC1401400).

## CONFLICT OF INTERESTS

The authors declare that they have no conflict of interest
